# Differential Impact of Acute High-Intensity Exercise on Circulating Endothelial Microparticles and Insulin Resistance between Overweight/Obese Males and Females

**DOI:** 10.1371/journal.pone.0115860

**Published:** 2015-02-24

**Authors:** Cody Durrer, Emily Robinson, Zhongxiao Wan, Nic Martinez, Michelle L. Hummel, Nathan T. Jenkins, Marcus W. Kilpatrick, Jonathan P. Little

**Affiliations:** 1 School of Health and Exercise Sciences, University of British Columbia, Okanagan Campus, Kelowna, British Columbia, Canada; 2 College of Education, University of South Florida, Tampa, Florida, United States of America; 3 Department of Kinesiology, University of Georgia, Athens, Georgia, United States of America; University of Birmingham, UNITED KINGDOM

## Abstract

**Background:**

An acute bout of exercise can improve endothelial function and insulin sensitivity when measured on the day following exercise. Our aim was to compare acute high-intensity continuous exercise (HICE) to high-intensity interval exercise (HIIE) on circulating endothelial microparticles (EMPs) and insulin sensitivity in overweight/obese men and women.

**Methods:**

Inactive males (BMI = 30 ± 3, 25 ± 6 yr, n = 6) and females (BMI = 28 ± 2, 21 ± 3 yr, n = 7) participated in three experimental trials in a randomized counterbalanced crossover design: 1) No exercise control (Control); 2) HICE (20 min cycling @ just above ventilatory threshold); 3) HIIE (10 X 1-min @ ∼90% peak aerobic power). Exercise conditions were matched for external work and diet was controlled post-exercise. Fasting blood samples were obtained ∼18 hr after each condition. CD62E^+^ and CD31^+^/CD42b^-^ EMPs were assessed by flow cytometry and insulin resistance (IR) was estimated by homeostasis model assessment (HOMA-IR).

**Results:**

There was a significant sex X exercise interaction for CD62E+ EMPs, CD31+/CD42b- EMPs, and HOMA-IR (all P<0.05). In males, both HICE and HIIE reduced EMPs compared to Control (P≤0.05). In females, HICE increased CD62E+ EMPs (P<0.05 vs. Control) whereas CD31+/CD42b- EMPs were unaltered by either exercise type. There was a significant increase in HOMA-IR in males but a decrease in females following HIIE compared to Control (P<0.05).

**Conclusions:**

Overweight/obese males and females appear to respond differently to acute bouts of high-intensity exercise. A single session of HICE and HIIE reduced circulating EMPs measured on the morning following exercise in males but in females CD62E+ EMPs were increased following HICE. Next day HOMA-IR paradoxically increased in males but was reduced in females following HIIE. Future research is needed to investigate mechanisms responsible for potential differential responses between males and females.

## Introduction

Individuals who are overweight or obese are at increased risk of developing insulin resistance (IR), type 2 diabetes (T2D), and cardiovascular disease (CVD) [1,2]. Exercise improves metabolic [3] and cardiovascular health [4], but the optimal exercise strategy for preventing T2D and CVD is not firmly established. Many of the benefits of exercise training are attributable to the acute effects of exercise, which can last for ∼24–48 h following each individual training session [5]. In particular, numerous investigations have shown that acute exercise improves insulin sensitivity for up to 24–48 h into recovery as assessed by fasting homeostasis model assessment (HOMA-IR) scores [6,7], oral glucose tolerance testing [8], and/or hyperinsulinemic-euglycemic clamp[9]. These findings have led to guidelines that recommend no more than one day off in between exercise sessions in individuals with, or at risk for, T2D [10].

Exercise also improves vascular health, with studies demonstrating improved endothelial function that can be measured for 2–72 h following a single bout of exercise [11–13]. Damage to, and dysfunction of, vascular endothelial cells is strongly associated with CVD risk and it is hypothesized that many of the cardiovascular benefits of exercise can be linked to improved endothelial cell function [4,14]. Insulin is a potent vasodilator, providing evidence that increased insulin sensitivity and vascular function following exercise may be linked, perhaps through enhanced delivery of glucose to skeletal muscle [15]. Therefore, examining how different exercise modalities acutely impact markers of insulin resistance and endothelial cell health may provide insight regarding the exercise option with the most therapeutic potential.

Although not all studies are in agreement, there is evidence that high-intensity exercise may lead to greater improvements in insulin sensitivity and endothelial function when compared to continuous moderate-intensity exercise [11,16–18]. Improvements in insulin sensitivity following acute high-intensity exercise may be linked to greater muscle fiber recruitment and/or muscle glycogen depletion, which augment skeletal muscle glucose uptake in the hours following exercise [19]. Vascular adaptations to exercise are mediated, at least in part, by shear stress acting on the endothelial cells that line conduit blood vessels [20]. In large conduit arteries, shear stress during and following exercise increases in direct proportion to intensity [21,22], suggesting that higher intensity exercise may have greater potential for improving endothelial function [23]. Cocks and colleagues have also shown that high-intensity training is comparable to traditional endurance-oriented exercise training for inducing microvascular adaptations [24], which could further improve insulin and glucose delivery to skeletal muscle to enhance insulin sensitivity.

High-intensity interval exercise (HIIE) has emerged as an exercise modality that is particularly effective at improving cardiometabolic health. A session of HIIE involves repeated bouts of vigorous intensity exercise that can last from a few seconds up to several minutes, separated by periods of rest or recovery. HIIE is potentially attractive as the rest periods can facilitate the completion of vigorous intensity exercise in clinical or unfit populations and overall training programs are time-efficient. As a result, HIIE has garnered attention in the general population and clinical settings as a potent, yet time-efficient, therapeutic exercise option [25,26]. Several training studies have compared HIIE to moderate-intensity continuous exercise and have demonstrated the HIIE is equal to, or superior, for improving markers of insulin sensitivity and endothelial function (for review see [27]). However, comparisons between HIIE and high-intensity continuous exercise (HICE) are sparse. It therefore remains to be determined how high-intensity exercise, when performed in an interval versus continuous fashion, impacts markers of insulin sensitivity and endothelial cell function.

Accordingly, the purpose of this study was to compare HIIE to HICE on markers of insulin resistance and endothelial damage. Since several of the benefits of exercise can be attributed to acute effects of the most recent bout of exercise [28] [5], we examined fasting glucose, insulin, and HOMA-IR as indicators of insulin resistance on the morning following an acute bout of HIIE, acute HICE matched for external work, and a no exercise control condition. To determine how these exercise interventions affected endothelial health we measured circulating endothelial microparticles (EMPs), which are membrane-bound vesicles released from endothelial cells in response to inflammatory activation and/or apoptosis [29]. EMPs are emerging as novel biomarkers of endothelial activation and damage that are increased in overweight/obese individuals at risk for CVD [30,31], have been shown to directly impair endothelial function [32], and are correlated with endothelial dysfunction in obesity [33]. We hypothesized that both types of exercise would improve insulin sensitivity and reduce EMP levels compared to the no exercise control condition but that responses to acute HIIE would be greater than acute HICE. Because insulin sensitivity and endothelial function may be different in males and females [34,35] an exploratory aim was to examine potential sex differences in the responses to acute HIIE and HICE.

## Materials and Methods

### Ethics Statement

The study protocol was approved by the University of South Florida Institutional Review Board / Human Research Protection Program. All participants provided written informed consent.

### Participants

Participants were 13 adults (6 male, 7 female, mean age ± SD = 23±4 years, mean BMI ± SD = 29 ± 3) at a large university in the southeastern United States. All participants were overweight or obese (BMI 25–35) and were inactive (defined as engaging in two or less bouts of physical activity per week [a bout was defined as purposeful moderate-to-vigorous intensity exercise lasting ≥20 minutes] [36]), but otherwise healthy. Descriptive characteristics of the males and females are shown in Table 1.

**Table 1:** Descriptive characteristics of the participants in the study.

	Males (n = 6)	Females (n = 7)	P-value
**Age (yr)**	24.5 ± 5.5	21.4 ± 2.6	0.22
**BMI (kg/m** ^**2**^)	30.2 ± 3.2	27.7 ± 2.2	0.13
**VO**_**2**_ **peak (ml/kg/min)**	28.7 ± 7.7	27.7 ± 3.9	0.75
**Peak Workload (W)**	234 ± 33*	178 ± 22	0.01

*P<0.05

### Study Design

After a baseline visit to complete written informed consent and medical screening to confirm the absence of orthopedic, cardiovascular, or pulmonary contraindications to exercise, participants completed a maximal exercise test, a familiarization session, and three experimental trials, each separated by at least 48 hours.

### Maximal Exercise Test

A progressive, ramp protocol was performed on an electronically-braked cycle ergometer (Lode, Groningen, Netherlands). The protocol ramp rate varied between 15–25 watts per minute and was based on a standardized formula [37]. The test was terminated when the participant could not maintain a pedal cadence of 30 rpm. Heart rate (HR), blood pressure (BP), ratings of perceived exertion (RPE), and expired gases were monitored using standard procedures. HR was measured using a HR monitor (Polar, Lake Success, NY) and BP was determined by auscultation. RPE was estimated each minute using the Borg CR-10 Scale [38]. Expired gases were collected through an air cushion mask and analyzed continuously using a metabolic cart (Vacumetrics, Ventura, CA). VO_2_ peak was identified as the largest volume of O_2_ consumed per minute during the test. Criteria for verifying maximal exertion were as follows: a peak HR of at least 90% of age-predicted maximal HR, a peak RPE of at least 9, and a peak respiratory exchange ratio (RER) of at least 1.15. VT was identified by visual inspection based on the ventilatory equivalents for oxygen and carbon dioxide [39].

### Familiarization Session

On a separate visit participants were familiarized to the laboratory procedures and experimental sessions. This allowed for confirmation and experience of the exercise intensities for the experimental trials, which were based on maximal testing data. Participants were also given a food recording sheet with instructions to record all food and drink consumed, and refrain from any exercise or alcohol for the 24 h period before their next visit.

### Experimental Exercise Trials

Upon arrival to the laboratory for the first experimental trial, the food record was reviewed by a researcher, photocopied, and returned the participant. Participants were given instructions to strictly follow the same diet for 24 h prior to each experimental trial, which was confirmed upon arrival to the laboratory. The participants completed three experimental trials in a randomized, counterbalanced fashion separated by at least 48 h. One trial served as a no exercise control (Control) and there were two high-intensity exercise trials that were matched for external work were performed. HICE involved 20 minutes of cycling at a power output corresponding to 10% of the difference between power at VT and peak power output (i.e., 10% delta, [40]). HIIE involved 12 X 1-min at a power output of 60% of the difference between VT and peak power output (i.e., 60% delta, [40]) interspersed with 1-min recovery periods at 10–20% of maximal capacity, based on calculations designed to ensure total work was equal for all trials. The HIIE protocol was based on previous research showing cardiometabolic benefits in individuals with obesity and type 2 diabetes [41,42] whereas the HICE protocol was chosen to ensure that it was vigorous exercise above the VT but tolerable and matched for work with HIIE. HR was monitored continuously throughout exercise to measure exercise intensity. Ratings of perceived exertion (RPE; Borg category-ratio [CR]-10 scale; REF) were assessed during every other interval and rest period in HIIE and at six corresponding timepoints during HICE. Average RPE for the entire exercise session was calculated. Both exercise trials included a two-minute warm-up and cool-down at ∼20% maximal capacity. After completing the trial the participants left the laboratory with their food records and were instructed to follow the exact same diet for the rest of day. Participants then reported to the laboratory the following morning after >10 h fast for collection of a blood sample by venipuncture. Blood was collected into EDTA tubes (BD Vacutainer) and plasma was collected by centrifugation at 1200 *g* for ten minutes. Samples were frozen at -80 C prior to analyses. For each participant, exercise and subsequent fasting blood samples were performed at the same time of day for all conditions.

### Blood analyses

**Endothelial microparticles (EMPs).** Circulating EMPs were measured in platelet poor plasma by flow cytometry following the methods of Jenkins et al. [43]. Frozen plasma samples were thawed at room temperature for 20 minutes and centrifuged at 1500g for 15 minutes at room temperature. The top two thirds of plasma were then further centrifuged at 1500g for another 15 minutes to obtain platelet-poor plasma. The top 100ul of platelet poor plasma was then incubated with fluorochrome labeled antibodies specific for CD62E (CD62E-phycoerythrin, BD Biosciences Cat. No. 551145), CD31 (CD31-V450, BD Biosciences, Cat. No. 561653), and CD42b (CD42b-APC, BD Biosciences, Cat. No. 551061) for 20 minutes in the dark at 4°C. Samples were then fixed with 93ul of 2% paraformaldehyde and diluted up to 500ul with sterile, 0.2um filtered, PBS and analyzed on a Miltenyi Biotec MACSQuant Analzyer. A microparticle size gate was determined using 900nm NIST Traceable polystyrene beads (Cat. No. 64019, Polysciences Inc., Warrington, PA, USA). Unstained and fluorescence minus one controls were used to differentiate between true events and background/debris. EMPs were identified as CD62E+ and CD31+/CD42b- events within the microparticle size gate.

**Plasma glucose and insulin.** Plasma glucose was measured by the hexokinase method on a clinical chemistry analyzer (Chemwell 2910, Awareness Technologies). Plasma insulin was measured by ELISA following the manufacturer’s protocol (Mercodia Ultrasensitive Insulin ELISA) with absorbance read on a microplate reader (iMark, Bio-Rad). All samples were run in duplicate. The coefficient of variation for duplicate samples was 2.8% for glucose and 6.8% for insulin. We utilized the homeostasis model assessment of insulin resistance (HOMA-IR), calculated by the HOMA2 Calculator (https://www.dtu.ox.ac.uk/homacalculator/download.php) to estimate insulin resistance [44]. This method is highly correlated (*r* = 0.7–0.9) with estimates of insulin sensitivity derived from the hyperinsulinemic–euglycemic clamp technique and minimal model analysis [45], and has been used in previous studies to detect significant improvements in insulin sensitivity on the morning following an acute bout of exercise [7,46].

### Statistical Analyses

Data was analyzed using SPSS v22. Normality was assessed using Q-Q plots and Kolmogorov–Smirnov test and data was log-transformed if required. Baseline differences for descriptive characteristics between sexes, as well as physiological parameters measured during the exercise trials, were assessed using an unpaired Student t-test. A two factor (sex X condition) ANOVA with repeated measures on the second factor was used to analyze EMPs, HOMA-IR, fasting glucose, and fasting insulin. Significant main effects and interactions were followed up with Fisher LSD post-hoc tests. Cohen’s d was used to calculated effect sizes on pairwise comparisons. Potential relationships between EMPs in the Control condition and the change in EMPs (difference between Control and HIIE and Control and HICE) were assessed using Pearson correlations. Significance was set at P ≤ 0.05.

## Results

### Exercise trials

All participants were able to complete the full HICE and HIIE bouts. Average HR (P = 0.007) and overall RPE (P = 0.03) during HICE was significantly higher (157 ± 16 beats•min^-1^; 82 ± 8% HRmax, RPE = 4.1 ± 0.9) than and during HIIE (149 ± 13 beats•min^-1^, 78 ± 6% HRmax, RPE = 3.5 ± 1.6,), which is likely a result of the rest periods built into HIIE.

### EMPs

**Fig 1:**
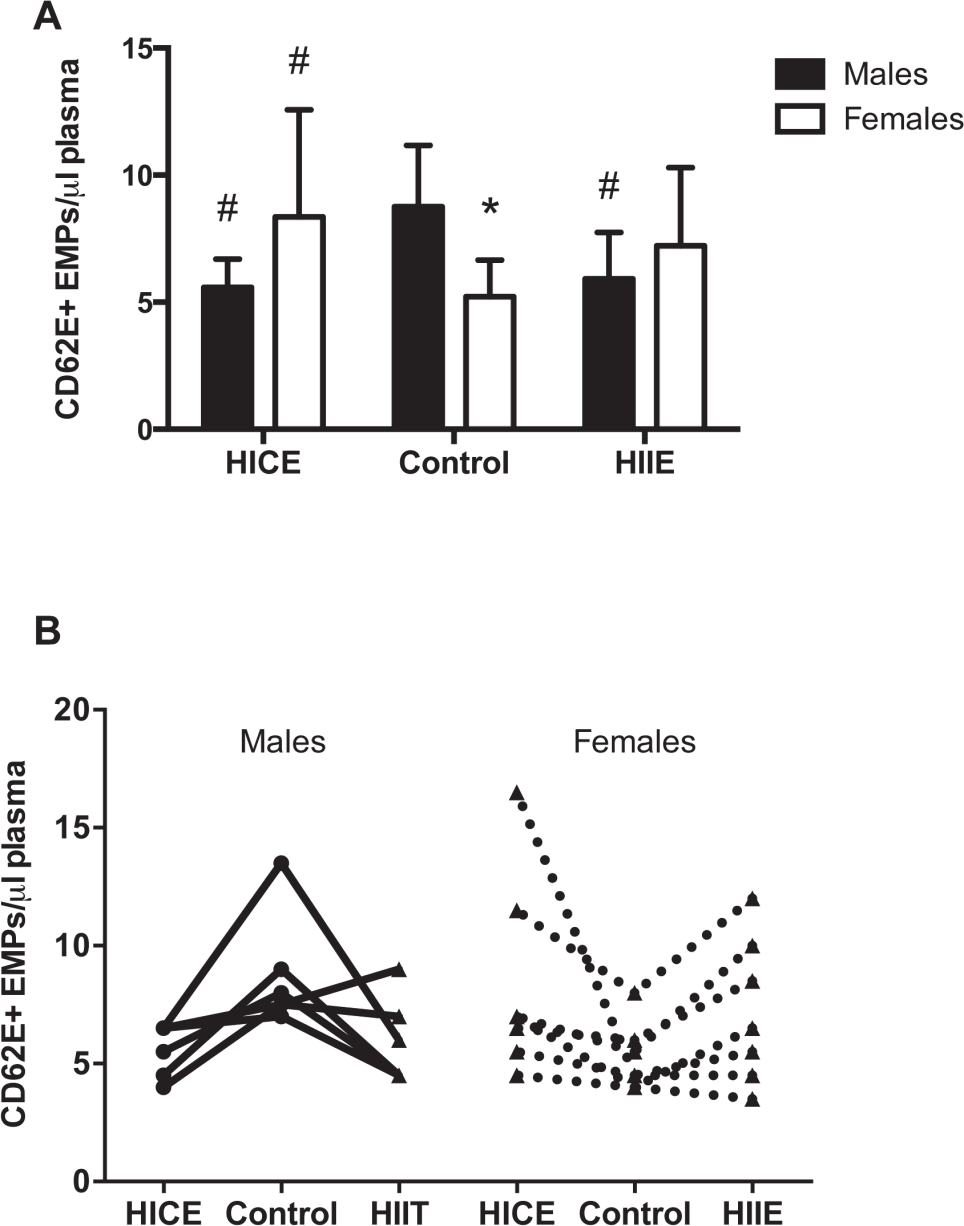
High-intensity exercise leads to sex specific changes in CD62E+ endothelial microparticles (EMPs). **A)** Fasting CD62E+ EMPs in males in females in the no exercise control (Control) condition and ∼18 hr following high-intensity continuous exercise (HICE) or high-intensity interval exercise (HIIE). **B)** Individual values for each male and female participant are shown connected by a line. The Control condition is shown in the middle to illustrate the effect of HICE or HIIE. *P<0.05 males vs. females. #P<0.05 vs. Control within sex.

There was a significant sex X condition interaction for CD62E+ EMPs (P = 0.008, Fig. 1A), indicating that males and females responded differently to exercise. Post-hoc analyses revealed that overweight/obese males had higher fasting levels of CD62E+ EMPs measured in the Control condition when compared to females (P<0.05). On the morning following both HICE and HIIE, fasting CD62E+ EMPs were reduced when compared to Control in the males (both P≤0.05, Cohen’s d = 1.68 for HICE and HIIE for 1.32) with no differences between HICE and HIIE (P>0.05). In females, HIIE resulted in an increase in fasting CD62E+ EMPs compared to Control measured on the morning following exercise (P<0.05, Cohen’s d = 1.00) with no differences between HICE and Control of HICE and HIIE (P>0.05). Individual responses are shown in Fig. 1B.

**Fig 2:**
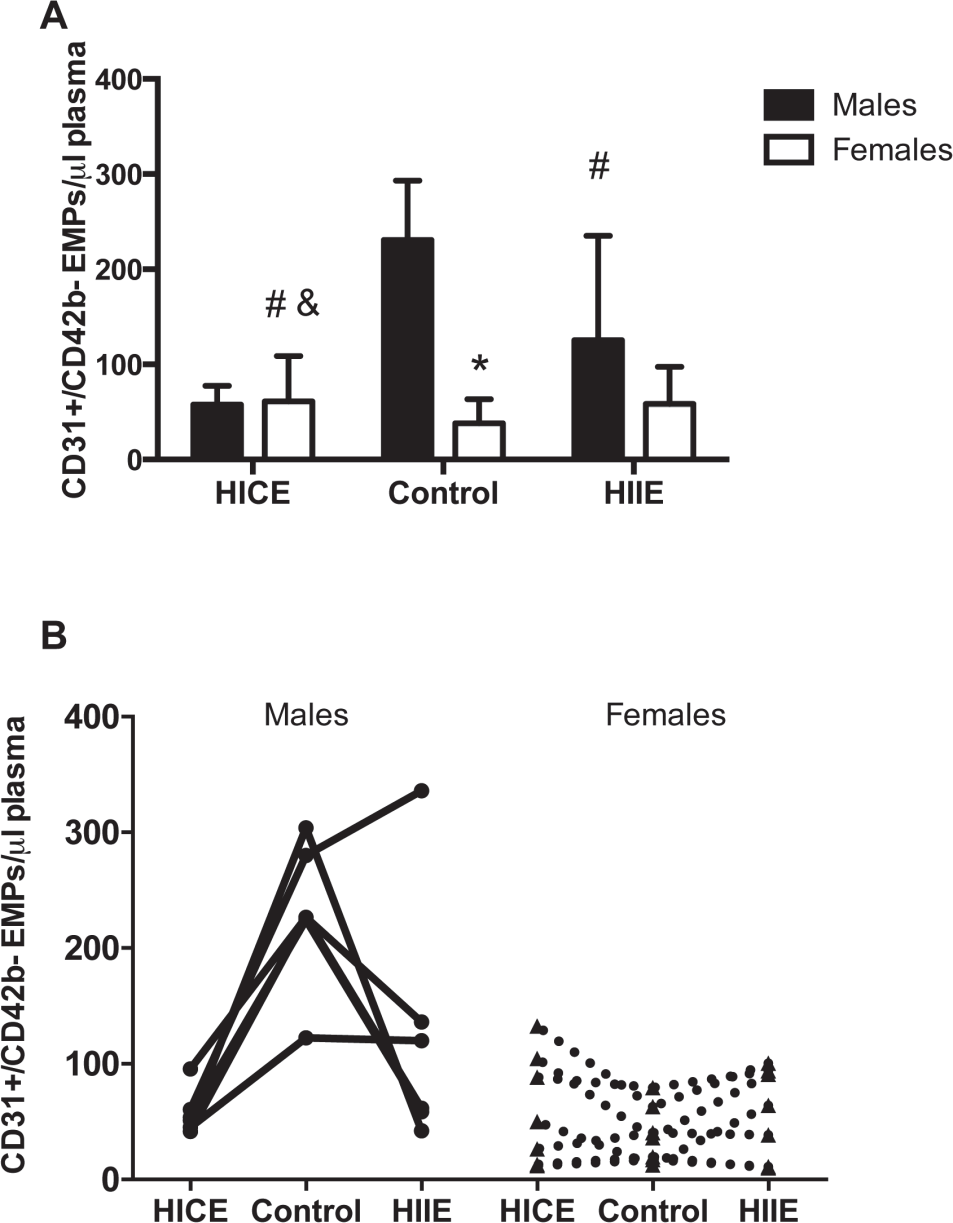
High-intensity exercise leads to sex specific changes in CD31+/CD42b- endothelial microparticles (EMPs). **A)** Fasting CD31+/CD42b- EMPs in males and females in the no exercise control (Control) condition and ∼18 hr following high-intensity continuous exercise (HICE) or high-intensity interval exercise (HIIE). **B)** Individual values for each male and female participant are shown connected by a line. The Control condition is shown in the middle to illustrate the effect of HICE or HIIE. *P<0.05 males vs. females. #P<0.05 vs. Control within sex. &P<0.05 vs. HIIE within sex.

There was also a significant sex X condition interaction for CD31+/CD42b- EMPs (P<0.001, Fig. 2A). Post-hoc analyses showed that overweight/obese males had higher levels of CD31+/CD42b- EMPs measured in the Control condition when compared to females (P<0.05). On the morning following both HICE and HIIE, fasting CD31+/CD42b- EMPs were reduced when compared to Control in the males (both P<0.05, Cohen’s d = 3.73 for HICE and 1.18 for HIIE) with HICE resulting in a greater reduction than HIIE (P<0.05, Cohen’s d = 0.86). In females, there were no significant differences between Control, HICE, or HIIE for CD31+/CD42b- EMPs.

**Fig 3:**
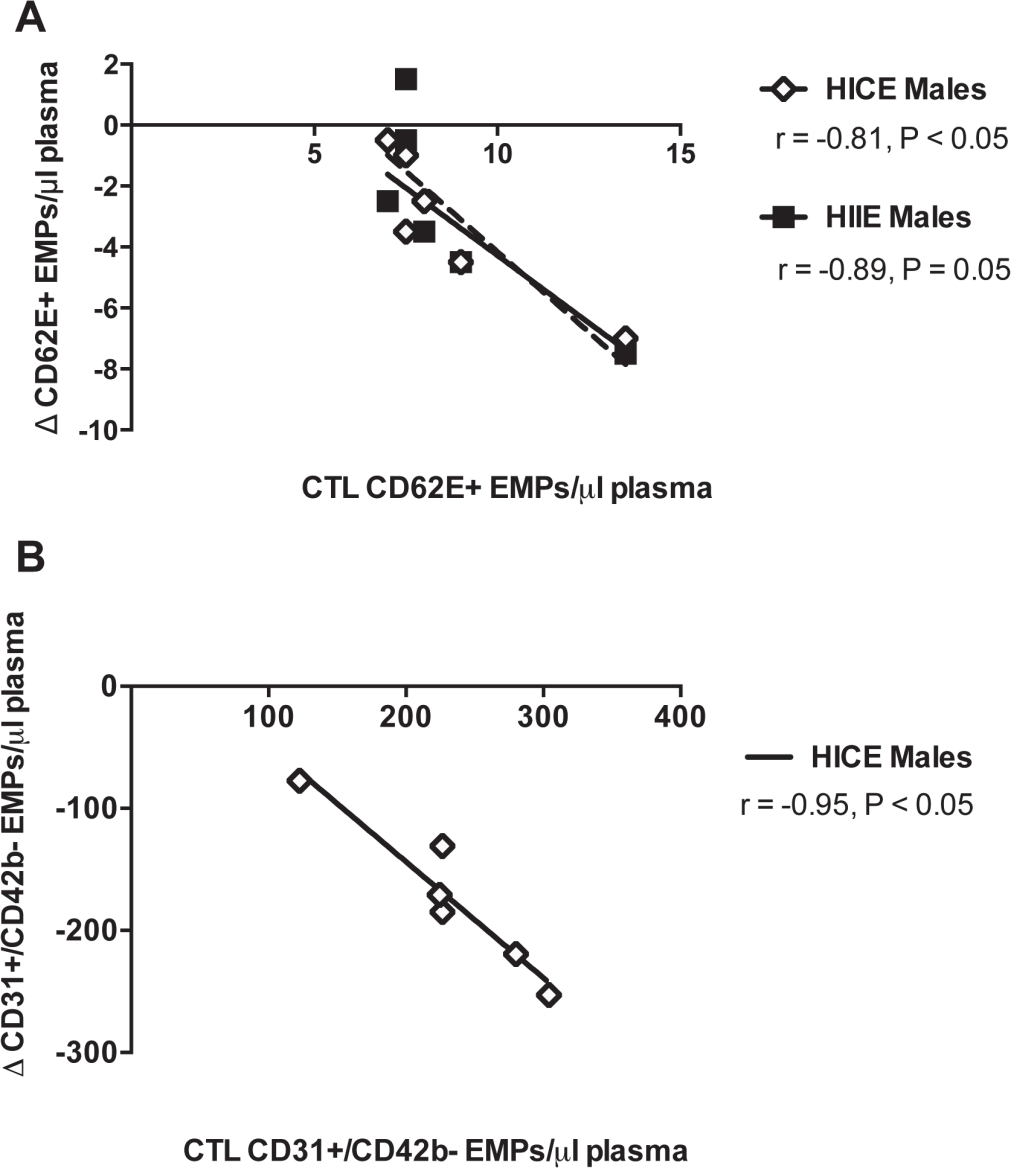
Relationships between endothelial microparticles (EMPs) measured in the sedentary Control condition and the changes seen after acute high-intensity exercise. **A)** Significant negative correlations were found for baseline CD62E+ EMPs measured in the no exercise control (Control) condition and the reduction seen after acute high-intensity continuous training (HICE) and high-intensity interval training (HIIE) in males. **B)** A significant negative correlation was found for CD31+/CD42b- EMPs measured in the no exercise control (Control) condition and the reduction seen after HICE in males.

To explore the relationship between baseline levels of EMPs measured in the Control condition and the changes in response to exercise, bivariate correlations were performed separately for males and females. In males, there was a significant negative correlation for CD62E+ EMPs such that those with the highest levels of CD62E+ EMPs measured in the Control condition experienced the largest reduction following HICE (r = -0.81, P = 0.02) and HIIE (r = -0.89, P = 0.05, Fig. 3A). In females, there was a tendency for a positive correlation between levels of CD62E+ EMPs measured in the Control condition and the increase seen following HIIE (r = 0.68, P = 0.09), with no significant correlation found for HICE (r = 0.084, P = 0.86).

For CD31+/CD42b- EMPs, levels measured in the Control condition were negatively correlated with the reduction seen following HICE (r = -0.95, P<0.001, Fig. 3B) but responses to HIIE were not significantly correlated to baseline levels (r = -0.38 P = 0.45). In females there were no significant relationships between Control levels of CD31+/CD42b- EMPs and responses to HICE (r = 0.09, P = 0.85) or HIIE (r = 0.22, P = 0.64).

### Plasma glucose and insulin

**Fig 4:**
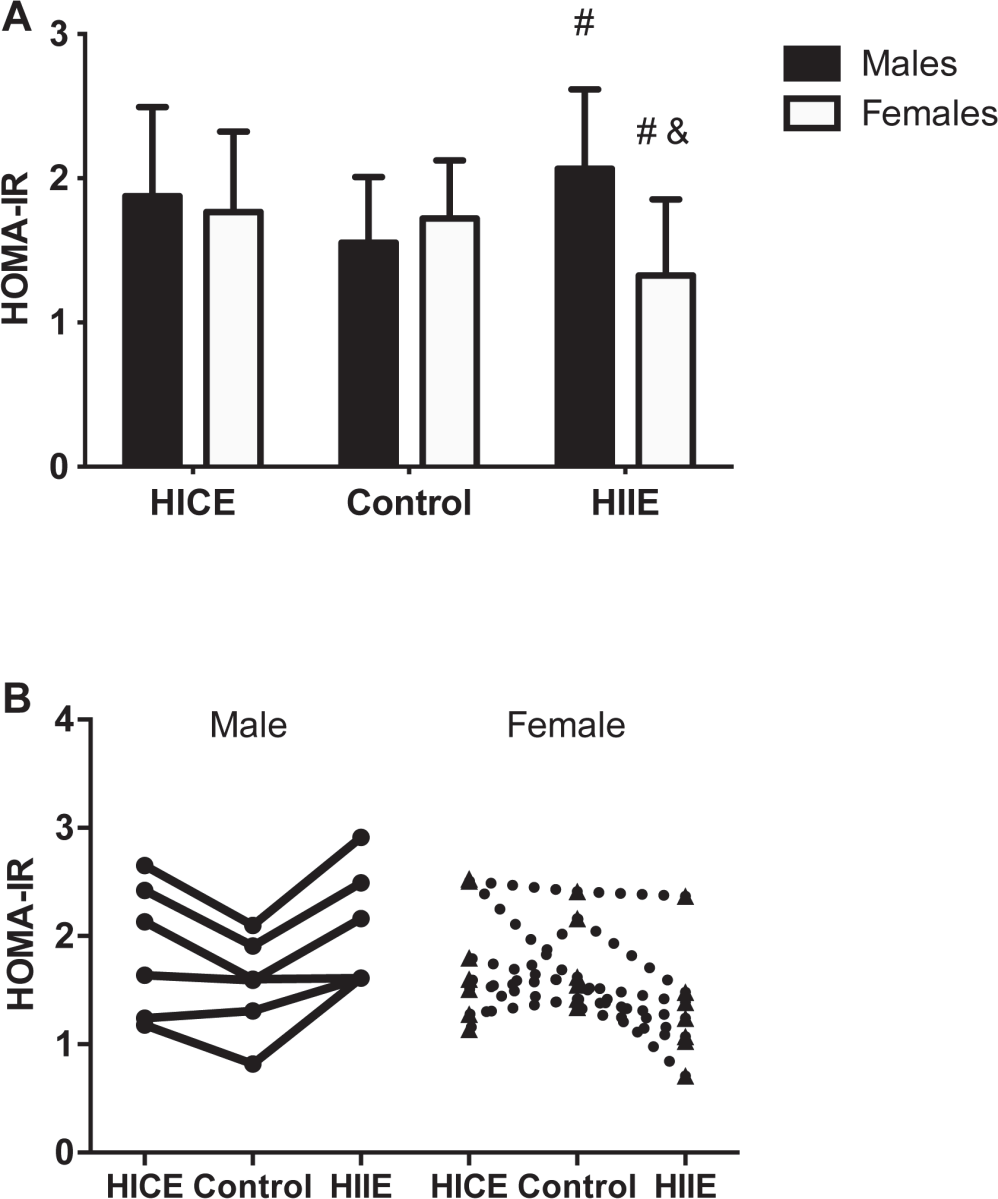
High-intensity exercise leads to sex specific changes in homeostasis model assessment of insulin resistance (HOMA-IR). **A)** Fasting HOMA-IR in males in females in the no exercise control (Control) condition and ∼18 hr following high-intensity continuous training (HICE) or high-intensity interval training (HIIE). **B)** Individual values for each male and female participant are shown connected by a line. The Control condition is shown in the middle to illustrate the effect of HICE or HIIE. #P<0.05 vs. Control within sex. &P<0.05 vs. HICE within sex.

There were no effects of sex (P = 0.11) or condition (P = 0.67) on fasting glucose (Table 2). However, analyses of fasting insulin revealed a significant sex X condition interaction (P = 0.002, Table 2). In males, HIIE resulted in a significant increase in fasting insulin when compared to Control (P<0.05) whereas in females HIIE resulted in a significant decrease in fasting insulin compared to both Control and HICE (both P<0.05). HOMA-IR followed the same pattern as fasting insulin, with a significant sex X condition interaction (P = 0.003, Fig. 4). Post-hoc testing revealed that HIIE led to an increase in insulin resistance in males when compared to Control (P<0.05, Cohen’s d = 1.01) but in females, HIIE led to a significant reduction in insulin resistance when compared to both Control and HICE (both P<0.05, Cohen’s d = 0.84 for Control and 0.81 for HICE).

**Table 2:** Fasting glucose and insulin in the sedentary control (Control), high-intensity interval training (HIIE) and high-intensity continuous training (HICE) conditions.

	Males			Females		
	Control	HIIE	HICE	Control	HIIE	HICE
**Fasting glucose (mmol/l)**	5.7±0.5	5.8±0.5	5.6±0.5	5.2±0.6	5.1±0.8	5.5±0.6
**Fasting Insulin (mmol/l)**	11.7±3.4	15.6±4.1*	14.2±4.6	13.3±2.7	10.2±3.8* #	13.4±4.0

*P<0.05 vs. Control.

#P<0.05 vs. HICE.

## Discussion

The main findings from this study are that circulating EMPs and fasting indices of insulin resistance appear to be responsive to acute high-intensity exercise in overweight and obese participants, but that apparent sex differences exist. In males, EMPs were reduced on the morning following a single bout of HIIE and HICE but HOMA-IR was paradoxically increased after HIIE. In contrast, EMPs were either unchanged (CD31+/CD42b-) or increased (CD62E+, for HICE only) in females whereas HOMA-IR was reduced on the morning following an acute bout of HIIE. These results, albeit in a small sample of overweight/obese young adults, indicate sex differences may exist when examining acute metabolic and vascular responses to high-intensity exercise. Our novel findings that high-intensity exercise can impact EMPs assessed the next morning also highlight the need to take into account recent exercise when assessing this proposed biomarker of endothelial cell health.

EMPs are membrane-bound vesicles released from endothelial cells in response to specific triggers. Based on findings in cell culture, CD62E+ EMPs have been shown to reflect endothelial activation or inflammation whereas CD31+/CD42b- EMPs are released upon endothelial cell apoptosis [29]. Circulating EMPs are increased in overweight/obese individuals with metabolic syndrome [32] and in various cardiovascular diseases [47]. Due to the benefits of exercise on endothelial function and recent evidence that traditional moderate-intensity endurance training can reduce EMP levels [48–50], we hypothesized that acute high-intensity exercise would lead to reduced EMPs in overweight/obese participants. Our results revealed a clear sex difference in EMP responses to exercise and this hypothesis was only supported for males.

Previous studies examining the impact of acute exercise on EMPs are limited and we are unaware of any studies in overweight or obese participants. Sossdorf and colleagues [51] reported a small increase in circulating EMPs in trained male subjects assessed 45 minutes after an acute bout of moderate-intensity cycling but levels returned to normal at two hours of recovery. In contrast, Chaar et al [52] found no effect of high-intensity exercise (three consecutive maximal exercise tests) on EMPs assessed up to 2 hr into recovery, although the authors reported undetectable EMP levels in their healthy subjects at all timepoints. Moreover, using a similar prior exercise model as employed in the current study Jenkins et al. [53] reported that both CD62E+ and CD31+/CD42b- EMP concentrations were significantly reduced ∼14–16 h after an acute bout of endurance exercise (45 min at ∼70% VO_2_ peak) when compared to a non-exercise control day in healthy young men. Only one study to our knowledge has examined EMP responses to HIIE. Guiraud et al. [54] measured EMPs after an acute bout of HIIE (40 X 15-sec at 100% Wpeak) and moderate-intensity continuous exercise (∼60% VO_2_ peak) in fit cardiac rehabilitation patients and reported no significant changes at 24 and 72 hours of recovery. However, the participants in this study were tested after completing several weeks of exercise training (including HIIE) and our participants were inactive prior to the experimental exercise trials. It is possible that exercise training, which is well known to improve endothelial function [4] and recently reported to reduce EMPs [48–50], may have reduced baseline EMPs in the fit cardiac rehabilitation patients studied by Guiraud and colleagues [54] such that acute exercise had limited effects. Future studies are needed to examine acute and chronic EMP responses to high-intensity exercise in individuals at elevated risk of cardiovascular disease. EMPs have been correlated with measures of flow-mediated dilation [33] and it will also be important to determine if EMP changes in response to exercise track measures of endothelial function.

The significant negative correlations between EMPs measured in the Control condition and the change in EMPs in response to HIIE and HICE for males were interesting. The data suggest that males with the highest baseline EMPs experience the largest reduction following high-intensity exercise. In females, there were no significant correlations found and if anything there was a tendency for a positive relationship between CD62E+ EMPs measured in the Control condition and the increase following HIIE. Although correlational analyses in small sample sizes are not without limitations, we feel these responses in our study further highlight sex differences in the EMP responses to acute high-intensity exercise.

To our knowledge, only one previous study has examined sex differences in EMPs. Toth and colleageus [55] reported increased EMPs in healthy young women compared to men, which is in contrast to our findings in young overweight/obese participants. It is possible that sex differences in EMPs manifest differently depending on body fatness or that obesity has greater effect on EMPs in males. It is generally accepted that pre-menopausal women are at a lower risk from cardiovascular disease when compared to men [56], which is attributed in part to a protective effect of estrogen. In as much that EMPs serve as a biomarker for endothelial cell health and cardiovascular risk [57], reduced EMPs in overweight/obese females in the present study supports a potential protective effect of estrogen on endothelial activation and/or damage.

The potential for exercise to improve insulin sensitivity is well established. Many studies have shown that a single bout of exercise can improve insulin sensitivity for up to 24 h following [6–8]. A series of studies form Magkos [6,7] has shown that HOMA assessed from fasting blood can detect next day improvements in insulin resistance. It has been shown that exercise energy expenditure [6], and possibly total duration of the exercise bout [58], are significant predictors of the improvements in insulin sensitivity following exercise. The majority of comparative studies have examined low/moderate-intensity versus high-intensity exercise preformed in a continuous fashion. Given the recent interest in HIIE for improving cardiometabolic health [23,25,41,59] we aimed to determine if the pattern of high-intensity exercise, performed as either HIIE or HICE, when matched for external work, impacted insulin resistance assessed by HOMA-IR on the morning following an acute bout. In females, HIIE reduced HOMA-IR when compared to both HICE and Control, indicating that a single session of HIIE may improve insulin sensitivity. The improvement in HOMA-IR following HIIE was driven by a reduction in fasting insulin as there were no changes in fasting glucose (Table 2). Interestingly, we recently reported that a single session of HIIE improved postprandial hyperglycemic responses to breakfast measured on the day following exercise in a sample of overweight/obese participants that were primarily female (8 females, 2 males) [42]. Taken together, these findings suggest that one session of HIIE can improve metabolic control on the day following exercise in overweight/obese females. In contrast to our hypotheses, neither HIIE nor HICE improved HOMA-IR in males. In fact, there was a significant increase in HOMA-IR on the morning following HIIE in males, which could be attributed to an increase in fasting plasma insulin. Whether this increase in HOMA-IR is reflective of a potential “negative” response to HIIE in overweight/obese males will require further investigation.

EMPs are generally considered to reflect vascular damage [47] and studies in mice have demonstrated that EMPs can directly impair endothelial function [32]. The contrasting findings with respect to markers of endothelial damage and insulin sensitivity in the present study (i.e., EMPs decreased while HOMA-IR increased in males, EMPs increase while HOMA-IR decreased in females) may reflect differences in the cardiovascular and metabolic effects of high-intensity exercise that are modulated by sex. However, some evidence now indicates that an increase in EMPs in response to cardiovascular stress may be a beneficial response [60] and that microparticles may be involved in angiogenesis or vascular remodeling [61]. Taken in this context, the increase EMPs and reduced HOMA-IR in females could be interpreted to reflect an adaptive response linked to the well-known cardiometabolic benefits of exercise.

The main limitation of the present study was that we were unable to procure blood samples prior to or at multiple time points after exercise and therefore could not determine the time course of EMP changes in response to acute exercise. However, the changes detected on the morning following exercise were quite robust and remarkably consistent across individual subjects (see Figs. 1B and 2B for individual values). The sample size in the present study was small, but analyses of the effect sizes for the differences between Control and both HICE and HIIE were all large (Cohen’s d = 0.84–3.7 for all pairwise comparisons that reached statistical significance for EMPs and HOMA-IR). Nonetheless, it will be important to conduct studies with larger sample sizes to further examine potential sex differences in the responses to high intensity exercise in overweight/obese males and females. It would also be of interest to examine integration between dynamic measures of insulin sensitivity, postprandial hyperglycemia, and EMPs on the morning following exercise but our measurements were limited to fasting samples only. In addition, our analyses were restricted to CD62E+ (inflammatory) and CD31+/CD42b- (apoptotic) microparticles. This was based on previous work in healthy humans that showed that these specific EMPs respond rapidly to acute alterations in blood flow [43]. However, EMPs can also express various other cell surface markers that may provide further insight into what triggered their release [47]. Addition of a measure of endothelial function (e.g., flow-mediated dilation) to see if changes in EMPs track functional markers of endothelial health would also have strengthened this study. We also did not strictly control for menstrual cycle within our female participants. This is a clear limitation but because the exercise trials were performed in a randomized and counterbalanced fashion 2–7 days apart, it seems highly unlikely that any systematic effects of menstrual cycle could have contributed to the differences reported in females in the present design.

In conclusion, this investigation demonstrates that overweight/obese males and females appear to respond differently to acute bouts of high-intensity exercise. A single session of HICE and HIIE reduced circulating EMPs measured on the morning following exercise in males but in females CD62E+ EMPs were increased following HICE. Next day insulin resistance paradoxically increased in males but was reduced in females following HIIE. Future research is needed to investigate mechanisms responsible for differential responses between males and females and to determine if sex differences exist in the optimal exercise strategy for improving cardiometabolic health.
